# Development of Chloroplast Microsatellite Markers and Assessment of Genetic Diversity and Population Structure of *Sophora tonkinensis* Gagnep. in Southwestern China

**DOI:** 10.3390/cimb48060562

**Published:** 2026-05-28

**Authors:** Xiaoyan He, Ying Liang, Chunli Wang, Xinghao Li, Shuangshuang Qin, Linxuan Li, Guili Wei, Danfeng Tang, Zhanjiang Zhang, Fan Wei

**Affiliations:** 1 Guangxi Key Laboratory of Medicinal Resources Protection and Genetic Improvement, Guangxi Engineering Research Center of TCM Resource Intelligent Creation, National Center for TCM Inheritance and Innovation, Guangxi Botanical Garden of Medicinal Plants, Nanning 530023, China; 15779034642@163.com (X.H.); 198yingzi@163.com (Y.L.); qin_double@126.com (S.Q.); lilinxuan1125@163.com (L.L.); weigl1992@163.com (G.W.); tdfmanuscript@163.com (D.T.); 2 School of Traditional Chinese Pharmacy, China Pharmaceutical University, Nanjing 211198, China; 3 Guangxi TCM Resources General Survey and Data Collection Key Laboratory, Guangxi Botanical Garden of Medicinal Plants, Nanning 530023, China; wangcli0318@163.com (C.W.); lxhao16@163.com (X.L.)

**Keywords:** *Sophora tonkinensis*, chloroplast SSR, chlorotype, seed-mediated dispersal, maternal lineage

## Abstract

*Sophora tonkinensis* Gagnep. is an important medicinal shrub native to the karst regions of southwestern China, where long-term overharvesting and habitat fragmentation have markedly reduced wild resources. Although recent phytochemical, transcriptomic, and chloroplast genomic studies have improved understanding of this species, its maternally inherited population structure has remained unclear. To address this gap, we developed nine novel chloroplast simple sequence repeat (cpSSR) markers and used them to genotype 274 individuals from eighteen wild populations. A total of 41 alleles were detected, with 2–10 alleles per locus, indicating moderate to high polymorphism at the species level. By combining the nine cpSSR loci, we further identified 25 chlorotypes, including 19 private chlorotypes. Within-population chloroplast diversity was generally low, and five populations were monomorphic, whereas HJSE and LYNG retained comparatively high chlorotype diversity. Genetic differentiation among populations was extremely strong (mean *FST* = 0.808), whereas historical gene flow was very limited (*Nm* = 0.112), and AMOVA showed that 85% of total chloroplast variation occurred among populations. Taken together, chlorotype network analysis, chlorotype geographic distribution, UPGMA, PCoA, and exploratory STRUCTURE analysis supported three geographically structured chloroplast groups, indicating long-term restriction of seed-mediated dispersal across the fragmented karst landscape. These newly developed cpSSR markers and the derived chlorotype framework provide a practical basis for tracing maternal lineages, prioritizing conservation units, guiding ex situ germplasm sampling, and informing future breeding of this nationally protected species. Overall, the present results describe chloroplast-based maternal structure rather than total genome-wide diversity in *S. tonkinensis*.

## 1. Introduction

*Sophora tonkinensis* Gagnep. (Fabaceae) is a perennial medicinal shrub distributed mainly in the limestone-dominated karst regions of southwestern China and northern Vietnam. Its dried roots, traded as Shan-dou-gen, are widely used in traditional Chinese medicine for inflammatory disorders of the throat and upper respiratory tract. Modern phytochemical and pharmacological studies further indicate that the species is rich in quinolizidine alkaloids, flavonoids, and related bioactive metabolites with anti-inflammatory, antiviral, antioxidant, and antimicrobial activities [1,2]. However, long-term destructive root harvesting, slow natural regeneration, habitat fragmentation, and the ecological fragility of karst systems have collectively intensified the decline of wild resources [3,4]. These pressures are compounded by cultivation constraints and the growing need to balance medicinal utilization with conservation of this nationally protected plant resource in China [5,6].

Despite its medicinal importance, population-level genomic resources for *S. tonkinensis* are still limited. In recent years, multi-omics studies have improved our understanding of tissue-specific alkaloid and flavonoid biosynthesis in this species [1,7]. Physiological and proteomic work has also provided useful information on seed dehydration tolerance and cryopreservation, which is relevant to ex situ conservation [8]. At the organellar level, the chloroplast genome of *S. tonkinensis* has been assembled and compared with related taxa, providing a valuable basis for marker development and species authentication [9,10]. Nevertheless, the only published population genetic study for wild *S. tonkinensis* based on AFLP markers reported only moderate nuclear differentiation, leaving maternal lineage structure and historical seed-mediated dispersal largely unresolved [11].

Chloroplast simple sequence repeats (cpSSRs) are particularly suitable for addressing this gap. In most angiosperms, plastid genomes are maternally inherited and effectively haploid, so cpSSR markers are highly informative for tracing maternal lineages, historical seed movement, and phylogeographic subdivision [12,13]. Because chloroplast genomes have smaller effective population sizes than nuclear genomes, plastid markers are generally more sensitive to genetic drift, bottlenecks, and long-term geographic isolation [14,15]. Recent studies have demonstrated the utility of newly developed cpSSR panels in medicinal, endangered, and geographically structured plants, including *Physalis angulata*, *Helichrysum italicum*, *Cryptomeria japonica* var. *sinensis*, *Orchidantha chinensis*, *Paeonia suffruticosa*, and *Sophora toromiro* [16,17]. Similar evidence from comparative plastome and cpSSR studies in *Allium*, *Lysionotus*, and *Quercus* further confirms the value of chloroplast variation for marker development, lineage discrimination, and conservation-oriented genetic assessment [18,19].

This approach is especially relevant for *S. tonkinensis* because it occupies patchy karst limestone habitats in which populations are often separated by deep valleys, rocky outcrops, edaphic heterogeneity, and expanding human land use. Karst ecosystems are widely recognized as centers of habitat fragmentation, microenvironmental heterogeneity, and localized evolutionary divergence, all of which can strengthen spatial genetic structure and limit seed-mediated connectivity [20,21]. Recent genomic and population studies of karst or limestone-associated plants, such as *Platycarya*, *Heteroplexis*, *Garcinia paucinervis*, *Liriodendron chinense*, and *Oreocharis mileensis*, similarly show that topographic complexity, ecological specialization, and anthropogenic disturbance can promote strong differentiation and conservation vulnerability [3,22]. Therefore, reduced seed dispersal and long-term habitat discontinuity are expected to enhance chloroplast lineage divergence in wild *S. tonkinensis* populations [23,24].

Analytical frameworks integrating diversity indices, differentiation statistics, Bayesian clustering, molecular variance, and multivariate ordination are well suited for testing whether maternally inherited variation is strongly structured across the species’ range [25,26]. Beyond population description, carefully curated chloroplast datasets can illuminate the spatial history of fragmented plant lineages and provide an evidence-based foundation for conservation prioritization, ex situ sampling, seed banking, and lineage-aware translocation in fragile karst systems [27,28]. In the present study, we developed nine novel cpSSR markers from the chloroplast genome of *S. tonkinensis* and used them to genotype 274 individuals from 18 wild populations in southwestern China. Our objectives were to: (1) evaluate the polymorphism and informativeness of the newly developed loci; (2) quantify chloroplast diversity within and among populations; (3) reconstruct chlorotypes and characterize maternal lineage structure and geographic differentiation; and (4) identify priorities for future conservation and targeted germplasm sampling.

## 2. Materials and Methods

### 2.1. Plant Materials and Sampling Strategy

A total of 274 individuals of *S. tonkinensis* were sampled from eighteen wild populations distributed across Guangxi and adjacent regions of southwestern China. The populations were designated DALG, DBMA, DLSN, FSCD, FSLJ, GZZY, HJML, HJSE, LCJA, LLTS, LYLX, LYNG, MSGL, MSLD, NPDL, TEWY, YZBS, and YZDS. Within each population, 6–22 adult individuals were sampled at intervals of at least 20 m to minimize the likelihood of collecting closely related plants. Young leaves were dried immediately in silica gel and stored until DNA extraction. Voucher specimens representing all populations were deposited in the herbarium of the Guangxi Botanical Garden of Medicinal Plants. The geographic locations of the sampled populations are shown in Table 1.

### 2.2. DNA Extraction

Total genomic DNA was extracted from approximately 50 mg of silica-dried leaf tissue using a modified cetyltrimethylammonium bromide (CTAB) protocol optimized for tissues rich in secondary metabolites. Leaf material was ground to a fine powder in liquid nitrogen and incubated in preheated extraction buffer containing 2% CTAB, 100 mM Tris-HCl (pH 8.0), 20 mM EDTA, 1.4 M NaCl, and 1% polyvinylpyrrolidone. DNA was purified with chloroform:isoamyl alcohol (24:1), precipitated with cold isopropanol, washed with 70% ethanol, and dissolved in TE buffer. DNA quantity and purity were assessed spectrophotometrically, and integrity was verified by 1.0% agarose gel electrophoresis. All samples were then diluted to approximately 20 ng/μL for PCR amplification.

### 2.3. cpSSR Marker Development and Primer Information

Candidate cpSSR loci were identified from the complete chloroplast genome sequences of *S. tonkinensis* [9,29] using standard microsatellite search criteria, with thresholds of 10 repeat units for mononucleotide motifs, five for dinucleotide motifs, four for trinucleotide motifs, and three for tetra- and pentanucleotide motifs. Primer pairs flanking candidate loci were designed with Primer Premier 5.0 and evaluated by in silico amplification against the chloroplast genome. Thirty primer pairs were synthesized and screened in a subset of samples. On the basis of amplification quality, reproducibility, and polymorphism, nine loci were retained for formal genotyping: StcpSSR01-StcpSSR09 (Table 2). Forward primers were labeled with FAM, HEX, TAMRA, or ROX to enable capillary fragment analysis.

### 2.4. PCR Amplification and Fragment Analysis

PCR amplification was performed in a 10 μL reaction system containing 5 μL of 2× Taq PCR Master Mix, 1 μL of mixed primers, 1 μL of DNA template, and 3 μL of ddH_2_O. The PCR reaction conditions were as follows: pre-denaturation at 95 °C for 5 min; denaturation at 95 °C for 30 s, annealing at a gradient of 62–52 °C for 30 s, and extension at 72 °C for 30 s for 10 cycles, with a decrease of 1 °C in each cycle; followed by denaturation at 95 °C for 30 s, annealing at 52 °C for 30 s, and extension at 72 °C for 30 s for 25 cycles; and a final extension at 72 °C for 20 min. Fluorescent PCR products were initially examined by agarose gel electrophoresis. Single bands of the expected size were selected and diluted to a comparable concentration range before capillary electrophoresis. Fragment analysis was then performed on an ABI 3730xl DNA Analyzer (Applied Biosystems, Foster City, CA, USA), and the raw fluorescence data were scored in GeneMarker v2.2.0.

### 2.5. Genetic Diversity and Population Structure Analyses

The raw data in .fsa format were exported from the ABI 3730xl DNA Analyzer, categorized according to locus, and imported into GeneMarker v2.2.0 software for genotype scoring. Genotypic raw data files and peak maps were then exported in Excel and PDF formats, respectively, by locus for subsequent analyses. Only clear, stable, and reproducible allele peaks were retained for downstream analyses, and loci with ambiguous peak patterns were rechecked manually in the electropherograms before final scoring.

Genetic diversity parameters, including the observed number of alleles (*Na*), the effective number of alleles (*Ne*), Shannon’s information index (*I*), observed heterozygosity (*Ho*), expected heterozygosity (*He*), and fixation index (*F*), were calculated using GenAlEx 6.5 [30]. Polymorphism information content (PIC) was calculated according to the method of Botstein et al. [31]. The degree of genetic differentiation and the calculation of genetic distance were also performed using GenAlEx 6.5. Because chloroplast cpSSR loci are effectively haploid and maternally inherited, heterozygosity- and fixation-related indices generated by the software were treated only as auxiliary descriptors, whereas biological interpretation focused primarily on chlorotype diversity, *FST*, AMOVA, and clustering patterns.

Principal coordinate analysis (PCoA) and analysis of molecular variance (AMOVA) were conducted in GenAlEx 6.5 [30] to evaluate genetic relationships among samples and partition molecular variation among populations, among individuals within populations, and within individuals. Statistical significance for AMOVA was tested using 999 permutations. For cluster analysis, an unweighted pair group method with arithmetic mean (UPGMA) tree was constructed using PHYLIP 3.69 based on the genetic distance matrix [32].

Population genetic structure was analyzed using STRUCTURE 2.3.4 [33] under an admixture model with correlated allele frequencies. Because all cpSSR loci are located on the same non-recombining chloroplast genome, STRUCTURE was used here only as an exploratory clustering tool rather than as a fully independent model-based inference equivalent to analyses based on unlinked nuclear loci. The number of clusters (*K*) was set from 1 to 20, and 20 independent runs was performed for each *K* value. The burn-in period and Markov chain Monte Carlo (MCMC) parameters were set to 10,000 and 100,000, respectively. The optimal *K* value was determined using the Δ*K* method of Evanno et al. [33] as implemented in Structure Harvester [34].

### 2.6. Chlorotype Reconstruction and Network Analysis

Each unique multilocus allelic profile across the nine cpSSR loci was treated as one chlorotype. Chlorotype frequencies were tallied for each population, and chlorotype diversity (*Hd*; Nei’s gene diversity) was calculated at the population level based on chlorotype frequencies. Private chlorotypes were defined as those detected in only a single population. Relationships among chlorotypes were visualized using a chlorotype network derived from pairwise multilocus cpSSR differences, and the geographic distribution of chlorotypes was mapped using the sampling coordinates of the 18 populations.

## 3. Results

### 3.1. Polymorphism of cpSSR Loci

We successfully amplified all nine cpSSR loci across the 274 individuals from the 18 sampled populations (Table 3). In total, the panel yielded 41 distinct alleles, ranging from 2 to 10 alleles per locus (mean *Na* = 4.556). Locus StcpSSR02 was the most polymorphic, displaying the highest effective number of alleles (*Ne* = 5.745), Shannon’s information index (*I* = 2.021), expected heterozygosity (*He* = 0.826), and polymorphism information content (PIC = 0.809). In contrast, StcpSSR03 was the least variable (*Ne* = 1.045; *I* = 0.106; *He* = 0.043; PIC = 0.042). Across all loci, the mean *Ne*, *I*, *He*, and PIC values stood at 2.593, 0.965, 0.487, and 0.451, respectively. Together, these metrics indicate a moderate to high level of chloroplast polymorphism at the species level. As expected for effectively haploid chloroplast markers, *Ho* values remained close to zero across loci. Accordingly, heterozygosity- and fixation-related indices are reported here only as software-derived descriptors, whereas the biological interpretation focuses primarily on *Hd*, *FST*, AMOVA, and clustering patterns.

Population differentiation statistics also indicated strong structuring of cpDNA variation (Table 4). The biologically informative result was the exceptionally high mean *FST* value of 0.808, indicating very strong differentiation among populations. Correspondingly, estimated historical gene flow (*Nm*) was consistently low across all loci, with a mean of only 0.112. These patterns suggest that chloroplast exchange among populations has been severely restricted, and that genetic drift has contributed substantially to chloroplast divergence across the fragmented landscape.

### 3.2. Genetic Diversity Within Populations

At the population level, chloroplast diversity was generally low (Table 5). The observed number of alleles (*Na*) ranged from 1.000 to 1.778. Five populations (DLSN, LLTS, MSGL, MSLD, and NPDL) were monomorphic across all loci (*Na* = 1.000; *He* = 0.000). Most remaining populations showed low diversity, with *He* values below 0.15. The main exceptions were DBMA and LYNG, which were the most variable populations in this study. DBMA showed the highest chloroplast diversity (*Na* = 1.667, *Ne* = 1.511, *I* = 0.417, *He* = 0.289), closely followed by LYNG (*Na* = 1.778, *Ne* = 1.401, *I* = 0.331, *He* = 0.209). As expected for chloroplast markers, the *Ho* in both of these populations remained zero.

### 3.3. Genetic Differentiation and Population Relationships

AMOVA revealed that 85% of the total cpSSR variation was partitioned among populations. The remaining 15% was attributable to differences among individuals within populations, whereas within-individual variation was effectively zero (Table 6).

**Figure 1:**
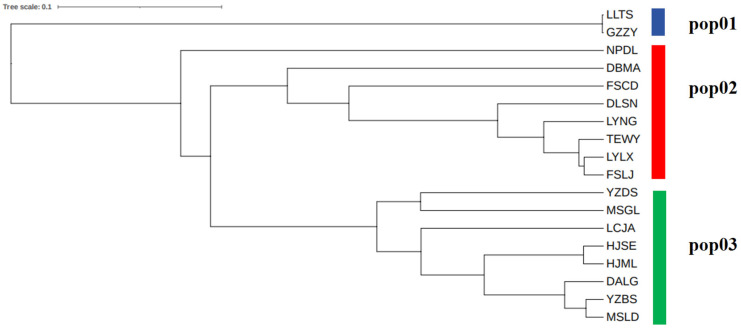
Dendrogram of eighteen *S. tonkinensis* populations based on UPGMA clustering analysis. Blue represents pop 1, red represents pop 2, and green represents pop 3.

Pairwise Nei’s genetic distances ranged from 0.001 between the closely related GZZY and LLTS, to 0.889 between NPDL and GZZY/LLTS (Table 7). The UPGMA dendrogram resolved these relationships into three major population groups (Figure 1): pop I grouped LLTS and GZZY; pop II clustered NPDL, DBMA, FSCD, DLSN, LYNG, TEWY, LYLX, and FSLJ; and pop III included YZDS, MSGL, LCJA, HJSE, HJML, DALG, YZBS, and MSLD.

### 3.4. Chlorotype Composition and Geographic Distribution

Based on the combined allelic profiles of the nine cpSSR loci, we identified 25 chlorotypes among the 274 sampled individuals (Table 8). Chlorotype frequencies were highly uneven across the dataset, with H1 (43 individuals) and H2 (41 individuals) the most frequent, followed by H3 (22 individuals), H4 (21 individuals), and H5 (19 individuals). A total of 19 chlorotypes were private, each occurring in only a single population, whereas only six chlorotypes were shared among two or more populations. Population-level chlorotype diversity was highest in HJSE (*Hd* = 0.762), LYNG (*Hd* = 0.711), DALG (*Hd* = 0.679), and FSLJ (*Hd* = 0.667), whereas DLSN, LLTS, MSGL, MSLD, and NPDL were monomorphic.

**Figure 2:**
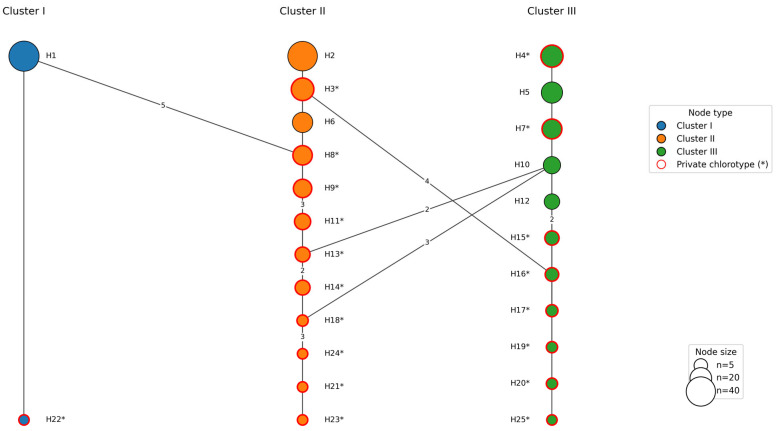
Chlorotype network based on multilocus cpSSR profiles. A chlorotype network was constructed using the combined allelic profiles of nine cpSSR loci from 274 individuals of *S. tonkinensis*. Each circle represents a chlorotype, and its area is proportional to the number of individuals harboring that chlorotype. Chlorotypes are arranged according to the three major chloroplast clusters identified in the population structure analyses (Cluster I, Cluster II, and Cluster III). Private chlorotypes occurring in only one population are marked with an asterisk and highlighted by red circle outlines. Numbers on branches indicate the number of mutational steps, defined here as the number of differing cpSSR loci between connected chlorotypes.

**Figure 3:**
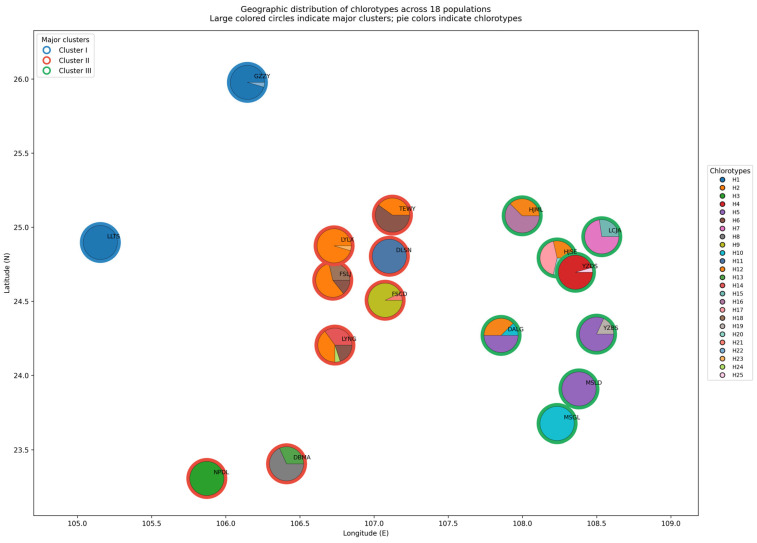
Geographic distribution of chlorotypes across 18 populations of *S. tonkinensis*. Pie charts represent the chlorotype composition of each population based on the combined allelic profiles of nine cpSSR loci, with pie colors indicating different chlorotypes (H1–H25). Large colored circles behind the pie charts indicate the three major chloroplast clusters identified in the population structure analyses, whereas the population codes are shown in black. Geographic coordinates correspond to the sampling locations listed in Table 1. This pattern reveals strong spatial structuring of maternally inherited chloroplast variation across the sampled range.

The chlorotype network and geographic distribution map revealed a clear subdivision into three major maternal lineage assemblages (Figure 2 and Figure 3). Cluster I was restricted to GZZY and LLTS and was dominated by H1. Cluster II included DBMA, DLSN, FSCD, FSLJ, LYLX, LYNG, NPDL, and TEWY and contained widespread chlorotypes H2 and H6 together with several private chlorotypes. Cluster III comprised DALG, HJML, HJSE, LCJA, MSGL, MSLD, YZBS, and YZDS and was characterized by H4, H5, H7, H10, and H12. No chlorotype was shared among the three major clusters, indicating strong spatial structuring of maternally inherited chloroplast variation and highly restricted seed-mediated gene flow.

### 3.5. Population Structure of S. tonkinensis Based on cpSSR Data

**Figure 4:**
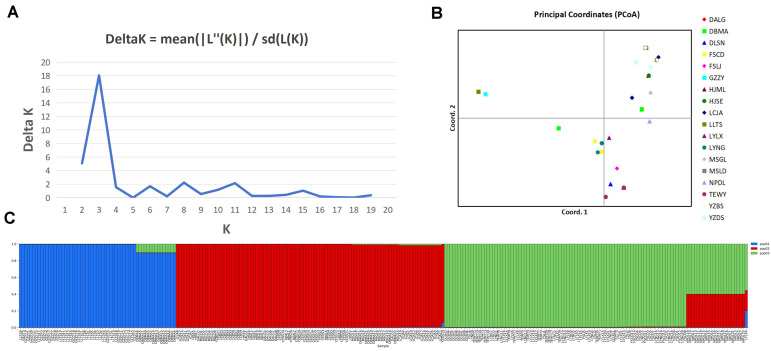
Population structure of *S. tonkinensis* inferred from cpSSR data. (**A**) Δ*K* values from STRUCTURE analysis; (**B**) principal coordinate analysis (PCoA) of sampled individuals; (**C**) assignment proportions of 274 individuals at *K* = 3. Different colors indicate the inferred chloroplast genetic groups.

STRUCTURE was used here as an exploratory clustering tool for the chloroplast dataset. A clear maximum Δ*K* occurred at *K* = 3, suggesting a three-cluster chloroplast pattern (Figure 4). This pattern was broadly consistent with PCoA and the assignment plot and, importantly, with the chlorotype network and geographic distribution results. The three clusters showed very limited admixture, further supporting strong maternal lineage differentiation across the sampled range of *S. tonkinensis*.

## 4. Discussion

### 4.1. Informativeness and Applicability of the Developed cpSSR Markers

The nine cpSSR loci developed here successfully provided sufficient resolution to characterize maternal variation across wild *S. tonkinensis* populations. Although the absolute number of loci is modest, the species-level allelic richness and PIC values indicate that this panel captures informative chloroplast polymorphism. Importantly, when the nine loci were combined into multilocus chlorotypes, they resolved 25 chlorotypes, including 19 private chlorotypes, thereby providing a direct framework for tracking maternal lineages. Recent comparative plastome work in *Sophora* and allied medicinal Fabaceae has further shown that chloroplast genomes harbor hypervariable regions and candidate DNA markers that are directly useful for species identification, phylogenetic inference, and conservation-oriented marker development in *S. tonkinensis* and related taxa [2,10]. Comparable marker performance has also been reported in newly developed cpSSR panels from *Physalis* and *Helichrysum* [16,17]. Additional studies in *Cryptomeria*, *Citrus*, *Orchidantha*, and *Paeonia* confirm that a moderate number of well-performing chloroplast loci can still provide strong discriminatory power in medicinal or geographically structured plants [25,26,35,36]. Similar conclusions from recent SSR-development studies in *Suaeda* and *Zanthoxylum* further support the practical value of compact but informative marker sets for diversity assessment and germplasm identification [25,37].

The observed variation in locus informativeness likely reflects the heterogeneous mutational behavior inherent to chloroplast simple repeats [16,25]. A/T-rich regions within plastid genomes often show uneven mutability, meaning that a small subset of highly variable loci can contribute disproportionately to lineage resolution. In our dataset, StcpSSR02 was especially informative, whereas lower-diversity loci still improved discrimination when considered jointly across the full geographic range [17,35]. Observed heterozygosity predictably approached zero across loci, which is consistent with the effectively haploid and usually maternally inherited nature of angiosperm chloroplast markers. For chloroplast cpSSR datasets, greater emphasis should be placed on chlorotype diversity, among-population differentiation, and lineage assignment than on heterozygosity-related summary statistics [26,36].

### 4.2. Strong Chloroplast Differentiation Reflects Restricted Seed-Mediated Gene Flow

One of the most striking findings of this study is the extraordinarily high level of chloroplast differentiation among wild populations of *S. tonkinensis*. The very high mean *FST* and the AMOVA result showing that most variation resides among populations point to severely restricted maternal connectivity. Similar strong partitioning of chloroplast variation has been reported in endangered or geographically structured taxa such as *Tetraena mongolica*, *Chimonobambusa utilis*, and *Bretschneidera sinensis* [12,14,15]. Chloroplast DNA phylogeography in bermudagrass likewise shows that maternal lineages can remain sharply partitioned across broad environmental and spatial gradients [38].

What drives this extreme isolation? Several non-exclusive ecological and historical processes may account for this pattern. One important factor is that *S. tonkinensis* is largely confined to discontinuous karst slopes and rocky outcrops, where valleys, exposed limestone, and human-modified land collectively act as strong barriers to seed movement. This interpretation is consistent with studies of limestone endemics and karst-adapted woody plants showing that habitat discontinuity, environmental filtering, and restricted dispersal promote deep lineage isolation [3,20]. Another likely contributor is the combination of environmental harshness, fragmentation, and demographic sensitivity in karst systems, which has also been emphasized for *Garcinia paucinervis* and other threatened karst plants [4,22]. In addition, molecular evidence from the karst-adapted genus *Primulina* indicates that high-calcium limestone habitats can drive adaptive differentiation, reinforcing the idea that local selection may accompany long-term geographic isolation [14].

The previous AFLP-based study of wild *S. tonkinensis* reported only moderate nuclear differentiation among populations [11]. By contrast, our cpSSR dataset revealed much stronger chloroplast structuring. This difference is biologically informative rather than contradictory, because nuclear markers reflect both pollen-mediated and seed-mediated gene exchange, whereas chloroplast markers mainly track maternally inherited seed dispersal. The much stronger spatial structure detected here therefore suggests that historical pollen flow has exceeded seed flow, while actual propagule movement across the karst landscape has remained highly constrained. Taken together, our results indicate that chloroplast variation preserves a deeper record of geographic isolation than was apparent from previously available nuclear-marker data.

### 4.3. Geographic Structuring and Possible Phylogeographic Implications

Together, the chlorotype network, chlorotype geographic distribution pattern, UPGMA, PCoA, and exploratory STRUCTURE analysis supported the presence of three major chloroplast groups. The chlorotype-based analysis provides the most direct evidence that these groups represent distinct maternal lineages within the sampled range of *S. tonkinensis*. Similar concordance among clustering and phylogeographic approaches has been reported in *Quercus* section *Cyclobalanopsis* and in comparative plastome studies of *Lysionotus*, both of which revealed clear geographic or lineage-associated chloroplast subdivision [19,23].

The geographic organization of these three lineages is ecologically plausible in light of the fragmented nature of karst environments. Genomic work on East Asian *Platycarya* indicates that adaptation to karst limestone and incipient speciation can be tightly linked in woody plants [3]. Comparative plastome studies in *Soroseris* and *Lysionotus* further show that heterogeneous mountain systems and restricted distributions can foster lineage persistence and divergence [19,39]. In our dataset, no chlorotype was shared among the three major groups, whereas shared chlorotypes occurred only within groups. Although the exploratory STRUCTURE analysis suggested limited admixture, the absence of chlorotype sharing across groups indicates that the principal chloroplast groups correspond to strongly differentiated maternal lineages rather than to a continuum of recent seed exchange.

### 4.4. Conservation Significance and Implications for Germplasm Management

The strong chloroplast structure documented here carries clear implications for the conservation of *S. tonkinensis*. Because a large majority of chloroplast variation resides between populations, the loss of any single local population could eliminate unique maternal diversity that is not recoverable elsewhere. Similar conservation warnings have been raised in recent genomic work on *Thuja sutchuenensis* and in nationwide assessments of ex situ conservation gaps for Chinese native flora [5,6].

From a practical standpoint, populations such as DBMA and LYNG warrant priority attention because they retain relatively high allele-level chloroplast diversity, while HJSE and DALG also deserve attention because they showed comparatively high chlorotype diversity. However, monomorphic populations should not be dismissed as unimportant. Even when internal variation is low, an isolated population may still represent a geographically distinctive lineage and thus contribute uniquely to the species-level chloroplast gene pool [13,15].

The new cpSSR markers developed here provide an operational tool for conservation-oriented sampling and germplasm management. For ex situ collections, maternal lines should be sampled systematically from multiple populations spanning all three chloroplast groups rather than collected opportunistically from a few accessible sites. Recent work on maximizing genetic representation in seed collections provides a useful framework for this strategy [27]. More broadly, restoration or translocation programs should avoid indiscriminate mixing of deeply differentiated chloroplast lineages, while monomorphic but geographically distinctive populations should still be retained to preserve lineage-level chlorotype diversity [22,28,40,41,42,43,44].

Accordingly, ex situ collections should include representative maternal lines from all three chloroplast groups, with priority sampling of DBMA, LYNG, HJSE, and DALG, while geographically distinctive monomorphic populations should also be retained.

## 5. Conclusions

Nine novel cpSSR markers revealed moderate chloroplast polymorphism at the species level but generally low chloroplast diversity within wild *S. tonkinensis* populations. When combined into multilocus chlorotypes, these markers resolved 25 chlorotypes, including 19 private chlorotypes, and clearly supported three major chloroplast lineage groups. Strong among-population differentiation indicated that maternal gene flow has been severely restricted across the fragmented karst landscape of southwestern China. Populations with relatively high chloroplast diversity, such as DBMA and LYNG, together with populations showing comparatively high chlorotype diversity, such as HJSE and DALG, should be prioritized for protection and ex situ sampling, while geographically distinctive low-diversity populations should also be conserved to preserve lineage-level variation. Overall, this study provides a practical chloroplast marker system and chlorotype framework for future conservation, breeding, and genomic research.

## Figures and Tables

**Table 1:** Sample sizes and location information for eighteen wild populations of *S. tonkinensis*.

No.	Population Code	Sample Size	Locations	Longitude (E)	Latitude (N)	Altitude (m)
1	DALG	8	Longma, Du’an, Hechi, Guangxi, China	107.8559	24.2696	622
2	DBMA	22	Ma’ai, Debao, Baise, Guangxi, China	106.4101	23.4058	1009
3	DLSN	9	Shuina, Donglan, Hechi, Guangxi, China	107.1029	24.8037	758
4	FSCD	14	Changdong, Fengshan, Hechi, Guangxi, China	107.0744	24.5069	750
5	FSLJ	7	Longjiang, Fengshan, Hechi, Guangxi, China	106.7215	24.6421	784
6	GZZY	22	Ziyun, Anshun, Guizhou, China	106.1462	25.9771	1217
7	HJML	8	Mulun, Huanjiang, Hechi, Guangxi, China	107.9983	25.0765	492
8	HJSE	7	Si’en, Huanjiang, Hechi, Guangxi, China	108.2337	24.7922	393
9	LCJA	22	Jian’ai, Luocheng, Hechi, Guangxi, China	108.5348	24.9355	670
10	LLTS	22	Tiansheng, Longlin, Baise, Guangxi, China	105.1548	24.8965	1416
11	LYLX	22	Luoxi, Leye, Baise, Guangxi, China	106.7306	24.8741	1129
12	LYNG	20	Nonggu, Lingyun, Baise, Guangxi, China	106.7361	24.2043	861
13	MSGL	10	Guling, Mashan, Nanning, Guangxi, China	108.2336	23.6760	506
14	MSLD	6	Lidang, Mashan, Nanning, Guangxi, China	108.3809	23.9088	510
15	NPDL	22	Delong, Napo, Baise, Guangxi, China	105.8726	23.3069	1026
16	TEWY	20	Wayao, Tian’e, Hechi, Guangxi, China	107.1235	25.0812	965
17	YZBS	11	Beishan, Yizhou, Hechi, Guangxi, China	108.4995	24.2782	302
18	YZDS	22	Desheng, Yizhou, Hechi, Guangxi, China	108.3573	24.6957	247

**Table 2:** Characteristics of nine cpSSR loci for PCR amplification in *S. tonkinensis*.

Primer No.	Primer Name	Fluorescent Label	Primer Sequence
StcpSSR01	StcpSSR01-F	HEX	AACAAAAACAAGCAAAACGGA
	StcpSSR01-R		AATTTCACACAACAGGGGGA
StcpSSR02	StcpSSR02-F	ROX	CCGAAACGAAACTACGGAAT
	StcpSSR02-R		AAAAAGGATTGAGCCGAATTT
StcpSSR03	StcpSSR03-F	ROX	TGTTTCACGTTTTCTGCCAA
	StcpSSR03-R		TCTCGTTCACCTCCAAAAAGA
StcpSSR04	StcpSSR04-F	HEX	TCCAATAACCATCCTTCCCTT
	StcpSSR04-R		GAGTTTTCACACCGGAAAGC
StcpSSR05	StcpSSR05-F	TAMRA	ACAGACCAAACTAAAGATATTTAGCAT
	StcpSSR05-R		GGGGGTCTGGCCTTATTTGA
StcpSSR06	StcpSSR06-F	FAM	GCCTTGATCCACTTGGCTAC
	StcpSSR06-R		TCGGGGTTTTAAAGTATACGAG
StcpSSR07	StcpSSR07-F	TAMRA	TGATGGGCATTCTTTGGTTT
	StcpSSR07-R		GCCAATTCGAATGACGAAAA
StcpSSR08	StcpSSR08-F	ROX	ACAGCGGATTTTCCAACAAG
	StcpSSR08-R		TTTCATCTGCACGAATGGTT
StcpSSR09	StcpSSR09-F	FAM	TCAAGCAGAGCCAAAAATTCTT
	StcpSSR09-R		ACCTCGATTTAATATTTGCACCTGA

**Table 3:** Genetic diversity parameters of nine cpSSR loci in *S. tonkinensis*.

Locus	*Na*	*Ne*	*I*	*Ho*	*He*	*F*	PIC	Prob	Signif
StcpSSR01	3.000	2.118	0.869	0.000	0.528	1.000	0.449	0.000	***
StcpSSR02	10.000	5.745	2.021	0.000	0.826	1.000	0.809	0.000	***
StcpSSR03	2.000	1.045	0.106	0.000	0.043	1.000	0.042	0.000	***
StcpSSR04	4.000	2.471	1.099	0.004	0.595	0.994	0.545	0.000	***
StcpSSR05	5.000	2.056	0.989	0.004	0.514	0.993	0.475	0.000	***
StcpSSR06	6.000	3.754	1.449	0.000	0.734	1.000	0.689	0.000	***
StcpSSR07	3.000	1.373	0.455	0.007	0.272	0.973	0.235	0.000	***
StcpSSR08	2.000	1.173	0.279	0.000	0.147	1.000	0.136	0.000	***
StcpSSR09	6.000	3.606	1.421	0.000	0.723	1.000	0.676	0.000	***
Mean	4.556	2.593	0.965	0.002	0.487	0.996	0.451	0.000	
STDEV	2.555	1.530	0.618	0.003	0.275	0.009	0.264	0.000	

Note: *Na*, number of observed alleles; *Ne*, effective number of alleles; *I*, Shannon’s information index; *Ho*, observed heterozygosity; *He*, expected heterozygosity; *F*, fixation index, a software-derived fixation index; PIC, polymorphic information content; Prob, *p*-value; Signif, significance (*** indicates extremely significant difference at *p* < 0.001).

**Table 4:** Population differentiation and historical gene flow estimates of nine cpSSR loci in *S. tonkinensis*.

Locus	*FST*	*Nm*
StcpSSR01	0.907	0.026
StcpSSR02	0.857	0.042
StcpSSR03	0.262	0.706
StcpSSR04	0.792	0.066
StcpSSR05	0.779	0.071
StcpSSR06	0.894	0.030
StcpSSR07	0.975	0.006
StcpSSR08	1.000	0.000
StcpSSR09	0.806	0.060
Mean	0.808	0.112
SE	0.073	0.075

Note: *FST*, genetic differentiation coefficient; *Nm*, historical gene flow estimate (*Nm* = 0.25(1 − *FST*)/*FST*).

**Table 5:** Genetic diversity indices of *S. tonkinensis* populations.

Population	*Na*	*Ne*	*I*	*Ho*	*He*	*F*
DALG	1.444 ± 0.242	1.225 ± 0.160	0.192 ± 0.112	0.000 ± 0.000	0.115 ± 0.068	1.000 ± 0.000
DBMA	1.667 ± 0.167	1.511 ± 0.128	0.417 ± 0.104	0.000 ± 0.000	0.289 ± 0.072	1.000 ± 0.000
DLSN	1.000 ± 0.000	1.000 ± 0.000	0.000 ± 0.000	0.000 ± 0.000	0.000 ± 0.000	1.000 ± 0.000
FSCD	1.444 ± 0.176	1.050 ± 0.022	0.091 ± 0.038	0.016 ± 0.010	0.045 ± 0.019	0.481 ± 0.200
FSLJ	1.222 ± 0.147	1.113 ± 0.080	0.112 ± 0.076	0.000 ± 0.000	0.073 ± 0.050	1.000 ± 0.000
GZZY	1.111 ± 0.111	1.005 ± 0.005	0.012 ± 0.012	0.005 ± 0.005	0.005 ± 0.005	−0.023 ± 0.005
HJML	1.222 ± 0.147	1.196 ± 0.130	0.147 ± 0.097	0.000 ± 0.000	0.104 ± 0.069	1.000 ± 0.000
HJSE	1.333 ± 0.167	1.230 ± 0.115	0.199 ± 0.100	0.000 ± 0.000	0.136 ± 0.068	1.000 ± 0.000
LCJA	1.333 ± 0.167	1.219 ± 0.110	0.195 ± 0.098	0.000 ± 0.000	0.132 ± 0.066	1.000 ± 0.000
LLTS	1.000 ± 0.000	1.000 ± 0.000	0.000 ± 0.000	0.000 ± 0.000	0.000 ± 0.000	1.000 ± 0.000
LYLX	1.333 ± 0.167	1.032 ± 0.016	0.062 ± 0.031	0.000 ± 0.000	0.029 ± 0.014	1.000 ± 0.000
LYNG	1.778 ± 0.324	1.401 ± 0.164	0.331 ± 0.132	0.000 ± 0.000	0.209 ± 0.083	1.000 ± 0.000
MSGL	1.000 ± 0.000	1.000 ± 0.000	0.000 ± 0.000	0.000 ± 0.000	0.000 ± 0.000	1.000 ± 0.000
MSLD	1.000 ± 0.000	1.000 ± 0.000	0.000 ± 0.000	0.000 ± 0.000	0.000 ± 0.000	1.000 ± 0.000
NPDL	1.000 ± 0.000	1.000 ± 0.000	0.000 ± 0.000	0.000 ± 0.000	0.000 ± 0.000	1.000 ± 0.000
TEWY	1.111 ± 0.111	1.103 ± 0.103	0.075 ± 0.075	0.000 ± 0.000	0.053 ± 0.053	1.000 ± 0.055
YZBS	1.222 ± 0.147	1.094 ± 0.062	0.105 ± 0.070	0.000 ± 0.000	0.066 ± 0.044	1.000 ± 0.000
YZDS	1.111 ± 0.111	1.005 ± 0.005	0.012 ± 0.012	0.005 ± 0.005	0.005 ± 0.005	−0.023 ± 0.005

Note: *Na*, number of observed alleles; *Ne*, effective number of alleles; *I*, Shannon’s information index; *Ho*, observed heterozygosity; *He*, expected heterozygosity; *F*, fixation index.

**Table 6:** Analysis of molecular variance (AMOVA) of populations.

Source	df	SS	MS	Est. Var.	%
Among Pops	17	1021.398	60.082	1.972	85%
Among Indiv	256	177.418	0.693	0.343	15%
Within Indiv	274	2.000	0.007	0.007	0%
Total	547	1200.816		2.323	100%

Note: SS, sum of squares; MS, mean square; Est. Var., estimated variance. Variation was partitioned among populations, among individuals within populations, and within individuals. The within-individual component was effectively zero, as expected for haploid chloroplast markers.

**Table 7:** Pairwise Nei’s genetic distance among *S. tonkinensis* populations.

Population	DALG	DBMA	DLSN	FSCD	FSLJ	GZZY	HJSE	LCJA	LLTS	LYLX	LYNG	MSGL	MSLD	NPDL	TEWY	YZBS	YZDS
DALG	-	0.497	0.516	0.443	0.485	0.778	0.100	0.224	0.778	0.456	0.508	0.215	0.047	0.523	0.518	0.048	0.295
DBMA	0.497	-	0.450	0.539	0.308	0.594	0.455	0.447	0.594	0.358	0.326	0.416	0.541	0.652	0.339	0.515	0.480
DLSN	0.516	0.450	-	0.295	0.137	0.667	0.556	0.477	0.667	0.119	0.110	0.444	0.556	0.556	0.152	0.556	0.557
FSCD	0.443	0.539	0.295	-	0.319	0.667	0.480	0.460	0.667	0.322	0.296	0.646	0.429	0.543	0.324	0.440	0.617
FSLJ	0.485	0.308	0.137	0.319	-	0.667	0.470	0.374	0.667	0.024	0.053	0.342	0.564	0.470	0.031	0.516	0.454
GZZY	0.778	0.594	0.667	0.667	0.667	-	0.778	0.699	0.001	0.646	0.667	0.778	0.778	0.889	0.667	0.778	0.761
HJML	0.110	0.442	0.556	0.472	0.476	0.778	0.024	0.189	0.778	0.447	0.500	0.289	0.197	0.468	0.509	0.166	0.290
HJSE	0.100	0.455	0.556	0.480	0.470	0.778	-	0.166	0.778	0.441	0.495	0.257	0.180	0.462	0.502	0.120	0.199
LCJA	0.224	0.447	0.477	0.460	0.374	0.699	0.166	-	0.699	0.358	0.402	0.292	0.292	0.461	0.407	0.244	0.293
LLTS	0.778	0.594	0.667	0.667	0.667	0.001	0.778	0.699	-	0.646	0.667	0.778	0.778	0.889	0.667	0.778	0.761
LYLX	0.456	0.358	0.119	0.322	0.024	0.646	0.441	0.358	0.646	-	0.083	0.312	0.534	0.450	0.029	0.487	0.425
LYNG	0.508	0.326	0.110	0.296	0.053	0.667	0.495	0.402	0.667	0.083	-	0.395	0.570	0.506	0.083	0.532	0.416
MSGL	0.215	0.416	0.444	0.646	0.342	0.778	0.257	0.292	0.778	0.312	0.395	-	0.333	0.556	0.374	0.286	0.223
MSLD	0.047	0.541	0.556	0.429	0.564	0.778	0.180	0.292	0.778	0.534	0.570	0.333	-	0.556	0.596	0.021	0.335
NPDL	0.523	0.652	0.556	0.543	0.470	0.889	0.462	0.461	0.889	0.450	0.506	0.556	0.556	-	0.485	0.508	0.557
TEWY	0.518	0.339	0.152	0.324	0.031	0.667	0.502	0.407	0.667	0.029	0.083	0.374	0.596	0.485	-	0.549	0.487
YZBS	0.048	0.515	0.556	0.440	0.516	0.778	0.120	0.244	0.778	0.487	0.532	0.286	0.021	0.508	0.549	-	0.240
YZDS	0.295	0.480	0.557	0.617	0.454	0.761	0.199	0.293	0.761	0.425	0.416	0.223	0.335	0.557	0.487	0.240	-

**Table 8:** Summary of chlorotypes identified from nine cpSSR loci in *S. tonkinensis*.

Chlorotype	No. of Individuals	No. of Populations	Populations Detected	Private Chlorotype
H1	43	2	GZZY, LLTS	No
H2	41	4	FSLJ, LYLX, LYNG, TEWY	No
H3	22	1	NPDL	Yes
H4	21	1	YZDS	Yes
H5	19	3	DALG, MSLD, YZBS	No
H6	17	3	FSLJ, LYNG, TEWY	No
H7	16	1	LCJA	Yes
H8	15	1	DBMA	Yes
H9	13	1	FSCD	Yes
H10	11	2	DALG, MSGL	No
H11	9	1	DLSN	Yes
H12	8	3	DALG, HJML, HJSE	No
H13	7	1	DBMA	Yes
H14	7	1	LYNG	Yes
H15	6	1	LCJA	Yes
H16	5	1	HJML	Yes
H17	3	1	HJSE	Yes
H18	2	1	FSLJ	Yes
H19	2	1	YZBS	Yes
H20	2	1	HJSE	Yes
H21	1	1	FSCD	Yes
H22	1	1	GZZY	Yes
H23	1	1	LYLX	Yes
H24	1	1	LYNG	Yes
H25	1	1	YZDS	Yes

## Data Availability

The original contributions presented in this study are included in the article. Further inquiries can be directed to the corresponding authors.
